# Effect of ethanol extracts of *Antrodia cinnamomea* on head and neck squamous cell carcinoma cell line

**DOI:** 10.1590/1414-431X20208694

**Published:** 2020-05-08

**Authors:** Li Liu, Chen Wang

**Affiliations:** 1 Anhui Medical College, Hefei, China; 2 Fuyang Vocational Technical College, Fuyang, China

**Keywords:** Antrodia cinnamomea, Head and neck squamous cell carcinoma cells, Proliferation, Invasion, Migration

## Abstract

Head and neck squamous cell carcinoma (HNSCC) is one of the most common malignant tumors. Ethanol extract of *Antrodia cinnamomea* (EEA) has been widely studied for its health benefits including anticancer effects. The purpose of this study was to assess the effects of EEA on HNSCC. Cell proliferation, transwell, and wound healing assays were performed. The impact of EEA on tumor growth was investigated using a xenograft model. Expressions of migration-related proteins (MMP-2, MMP-9, TIMP-1, and TIMP-2) and apoptosis-related proteins (cleaved caspase-9 and cleaved PARP) were determined using western blot analysis. The results indicated that EEA significantly inhibited the capacities of proliferation, invasion, and migration of HNSCC cells in a dose-dependent manner. Cleaved caspase-9 and cleaved PARP expressions were increased in cells treated with an increasing concentration of EEA, which suggested that EEA induced apoptosis of HNSCC. MMP-2 and MMP-9 were downregulated when cells were administered EEA, while TIMP-1 and TIMP-2 were not affected, which uncovered the mechanisms mediating the EEA-induced inhibition on cell invasion and migration. The animal experiment also suggested that EEA inhibited tumor growth. Our study confirmed the inhibitive effects of EEA on cell proliferation, invasion, and migration of HNSCC *in vitro* and *in vivo*, providing the basis for further study of the application of EEA as an effective candidate for cancer treatment.

## Introduction

Head and neck squamous cell carcinoma (HNSCC) is one of the most common malignant tumors, representing over 95% of all head and neck cancer cases (1,2). Despite advanced prevention and treatment strategies, thousands of new cases are reported annually (3), while treatment outcomes are still unsatisfactory with a five-year overall survival of about 50% (1). Most patients experience recurrence and poor prognosis after surgical resection, leading to a high mortality rate (4,5). Studies have revealed that the high recurrence rate is mainly due to enhanced invasion, migration, and proliferation capacities of tumor cells (6). The development of new chemotherapeutic agents for HNSCC is urgently needed.

*Antrodia cinnamomea* has been commonly used as a traditional Taiwanese medicine for its diverse applications in healthcare including anticancer effects (7). Previous studies have established its anti-tumor effects (1,8,9). The protective role of the ethanol extract of fruiting bodies of *Antrodia cinnamomea* has been experimentally demonstrated in several malignancies including renal cancer (10), lung cancer (7,11), hepatocellular carcinoma (12), and breast cancer (13), while the effects on head and neck cancer were seldom evaluated (14). Several active components isolated from raw extracts of *Antrodia cinnamomea*, mainly including triterpenoids, dicarboxylic acids, and polysaccharides (15), have been identified and can effectively inhibit proliferation, invasion, and migration of tumor cells (10,14). Moreover, extracts of *Antrodia cinnamomea* were reported to sensitize lung cancer cells and hepatic carcinoma cells to chemotherapeutics (9,12,13,16,17), indicating it can serve as a promising source of adjuvant agents combined with conventional therapy. However the potential application of *Antrodia cinnamomea* in treatment of HNSCC remains unclear.

In this study, we aimed to assess the potential effect of *Antrodia cinnamomea* on HNSCC cells.

## Material and Methods

### Ethanol extract of fruiting bodies of *Antrodia cinnamomea* (EEA)

Fruiting bodies of *Antrodia cinnamomea* were purchased from Cosmox Biomedical Co. LTD (Taiwan) and identified by the Bioresource Collection and Research Center (BCRC, Taiwan). EEA was prepared as described in a previous study (7) with minor modifications. Briefly, 300 g of dried fruiting bodies of *Antrodia cinnamomea* was soaked in 1 L ethanol for three days at room temperature. Then, the sample was filtered with filter paper and the filtrate was collected. The residue was soaked in 1 L of fresh ethanol again, the sample was filtered, and the filtrate was collected. All filtrate was pooled and concentrated to a paste by vacuum distillation using a rotary evaporator (N-11, EYELA; Japan). The extract was dissolved in dimethylsulfoxide (DMSO, Sigma-Aldrich Corp., USA) at a concentration of 50 mg/mL as a stock solution for further study.

### Cell culture

A tongue squamous cell carcinoma line (SAS) was purchased from the Type Culture Collection of the Chinese Academy of Sciences (China) and maintained in DMEM (HyClone, USA) supplemented with 10% fetal bovine serum (HyClone). Cells were cultured in a humidified incubator at 37°C under 5% CO_2_.

### Western blot analysis

Total cell protein was extracted using RIPA lysis buffer (Cell Signaling Technology, USA). Protein was determined by a Pierce BCA protein assay kit (Thermo Fisher Scientific, Inc., USA). Then, protein samples were resolved on 10% SDS-PAGE and transferred to polyvinylidene difluoride membranes. The membranes were blocked with 5% fat free milk for 1 h at room temperature, then incubated with the primary antibodies targeting human GAPDH (Cell Signaling Technology, Cat 8884), cleaved PARP (Cell Signaling Technology, Cat 5625), cleaved caspase-9 (Cell Signaling Technology, Cat 9505), MMP-2 (Cell Signaling Technology, Cat 40994), MMP-9 (Cell Signaling Technology, Cat 13667), TIMP-1 (Cell Signaling Technology, Cat 8946), or TIMP-2 (Cell Signaling Technology, Cat 5738) overnight at 4°C. After washing, the membrane was incubated with goat anti-rabbit (Cell Signaling Technology, Cat 7074) or rabbit anti-mouse (Cell Signaling Technology, Cat 7076) IgG H&L secondary antibody conjugated with horseradish peroxidase. Recommended dilutions for each antibody are listed in Table 1. The signal was developed using SuperSignal West Dura Extended Duration chemiluminescence substrate (Thermo Fisher Scientific, Inc.) and measured by ChemiDoc™ XRS+ System (Bio-Rad, USA). Three independent experiments were performed.

### Proliferation assay

For the cell proliferation assay, SAS cells were plated into a flat bottom 96-well plate at 2×10^3^ cells per well in triplicate with 100 µL DMEM (HyClone, USA) the day before and then cells were exposed to different concentrations of EEA. Cell counting kit-8 reagent (CCK-8, China) was added into each well after incubation at 37°C in a 5% CO_2_ atmosphere for 24, 48, 72, 96, and 120 h. After incubation for 2 h, absorbance at 450 nm was measured using a microplate reader (Bio-Rad). Three independent experiments were performed.

### Transwell and wound healing assays

A total of 4×10^4^ SAS cells in 100 μL serum-free DMEM medium (HyClone) supplemented with different concentrations of EEA were plated into the upper chamber of an 8-μm pore membrane (Corning, USA) coated with matrigel (Becton, Dickinson and Company, USA) and 600 μL complete medium was added as a chemoattractant to the lower chamber. After 36-h incubation, non-invading cells were scraped out with a cotton swab, membranes were then fixed with 100% methanol and stained with 0.5% crystal violet. Then, cells were photographed using a digital light microscope and five fields were selected randomly to evaluate the average cell number on each membrane. Three independent experiments were performed.

For the wound healing assay, 1×10^5^ cells were seeded in 6-well plates at 37°C. When confluence reached 100%, the monolayer of cells was scratched with a sterilized pipette tip so as to create a wound. Dead cells were then washed away with PBS and monolayer cells were treated with various concentrations of EEA in DMEM supplemented 10% FBS at 37°C for 36 h. Images were captured by a digital light microscope at 0 and 36 h. Migration rate of cells at 36 h was indicated as wound width normalized to that at 0 h. Three independent experiments were performed.

### Animal experiments

All animal experiments were approved by the Animal Care and Use Committee of Fuyang Vocational Technical College (FVTC-20180621-031J-029). All procedures were in accordance with the Animal Care and Use Committee of the institution and conformed to legal mandates and national guidelines for the care and maintenance of laboratory animals. A total of 12 nude BALB/c mice (females, 6–8 weeks) were purchased from SLAC Laboratory Animal Co. Ltd. (China). SAS cells (10 million) were subcutaneously injected into the left flank of nude BALB/c mice. When tumors were palpable, all mice were randomized into 2 groups of 6 mice each. EEA stock solution was diluted to 20 mg/mL using normal saline for animal treatment. One group was orally administered diluted EEA solution at a dose of 100 mg/kg every other day for three weeks, and the other group was treated with an equal volume of solvent (60% normal saline + 40% DMSO). Tumor volume was monitored every three days using a caliper. After 57 days, mice were sacrificed and tumors were excised. Tumor volume was calculated using the formula: volume = 0.5 × length × width^2^.

### Statistical analysis

All statistical analyses were performed using SPSS 19.0 (IBM, USA). Data are reported as means±SD. The *t*-test was used to compare the differences between experimental and control groups, while one-way ANOVA was used when more than two groups were compared. P<0.05 was considered to indicate a statistically significant difference.

## Results

### EEA effectively inhibited the proliferation of HNSCC cells

**Figure 1:**
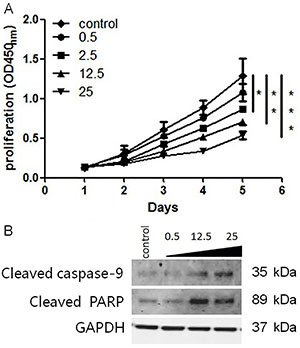
Ethanol extracts of *Antrodia cinnamomea* (EEA) inhibited cell proliferation in a dose-dependent manner. **A**, SAS cells were treated with EEA ranging from 0.5 to 25 μg/m, and proliferation inhibition was significantly observed in cells treated with EEA over 2.5 μg/mL after incubation for 5 days. **B**, Cleaved caspase-9 and cleaved PARP were increased in cells treated with EEA. Data are reported as means±SD. *P<0.05, **P<0.01, ***P< 0.001 (ANOVA).

We first assessed the effect of EEA on the proliferation capacity of HNSCC cells. Human tongue carcinoma cells (SAS) were treated with EEA ranging from 0.5 to 25 μg/mL. After incubation for 5 days, cell proliferation was significantly inhibited by EEA (2.5 to 25 μg/mL) compared to control, and a dose-dependent effect was also observed (Figure 1A). To further investigate the inherent molecular mechanisms mediating the inhibition effect of EEA on HNSCC cell proliferation, western blot analysis was conducted to determine the cellular levels of cleaved caspase-9 and cleaved PARP, which were markedly increased in cells treated with EEA in a dose-dependent manner (Figure 1B). Increased cellular levels of cleaved caspase-9 and cleaved PARP have been well-known as markers for the starting of cell apoptosis. Based on the above data, it can be hypothesized that EEA might effectively inhibit HNSCC cell proliferation potentially via provoking cell apoptosis.

**Figure 2:**
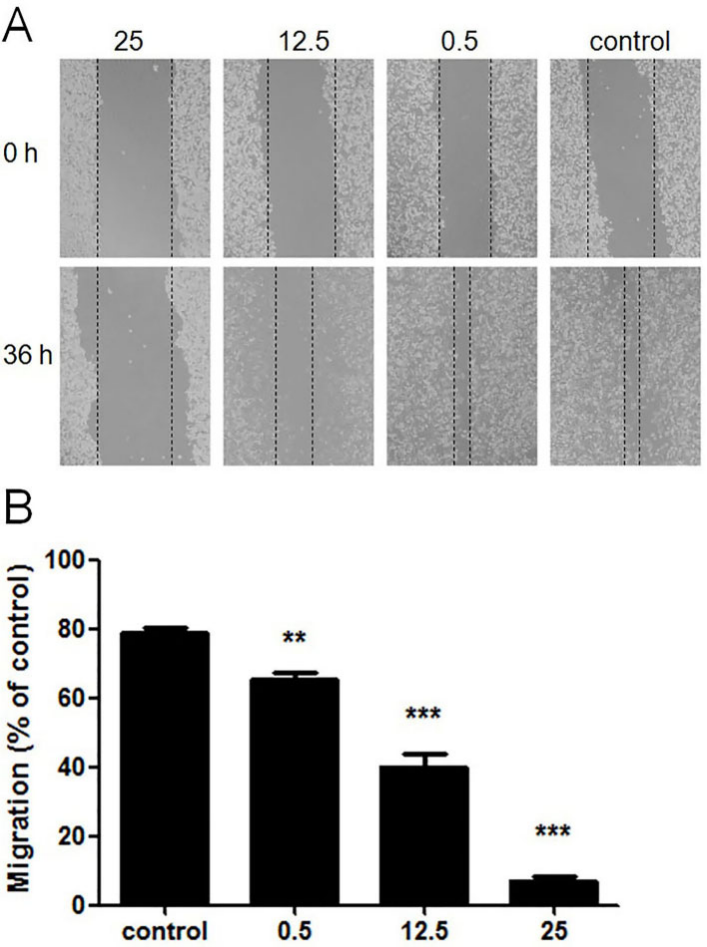
Suppression of wounding healing by ethanol extracts of *Antrodia cinnamomea* (EEA). **A**, A wound was made when cell confluence was 100% and fresh medium supplemented with different concentrations of EEA was added. Images were captured at 0 and 36 h. **B**, Migration distance was calculated and normalized to control that was not treated with EEA. Data are reported as means±SD. **P<0.01, ***P<0.001 compared to control (ANOVA).

**Figure 3:**
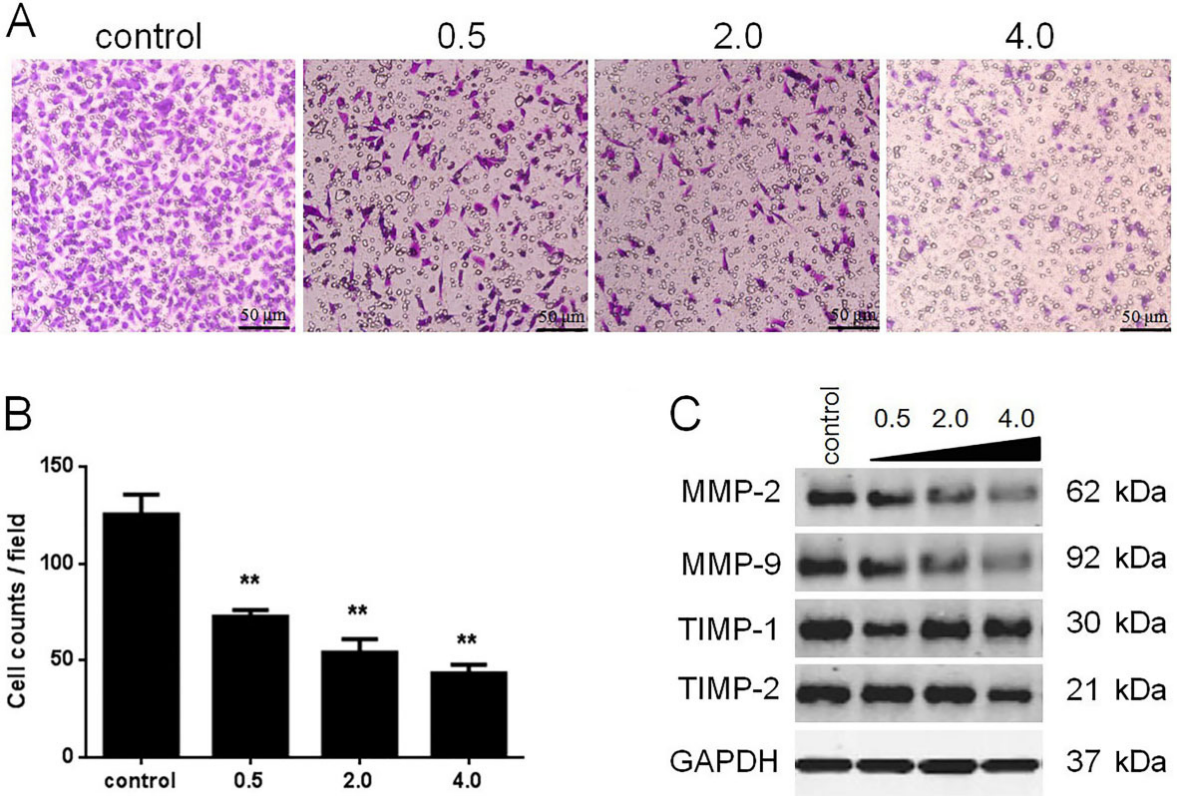
Inhibitive effects of ethanol extracts of *Antrodia cinnamomea* (EEA) on SAS cell invasion. **A**, SAS cells were plated into the upper chamber in medium supplemented with various concentrations of EEA. After 36-h incubation, invading cells were counted in five randomly selected fields (scale bars: 50 μm. **B**, The average counts of invading cells were significantly less in groups treated with EEA than in control. **C**, Effects of EEA on invasion-associated proteins, including MMP-2, MMP-9, TIMP-1, and TIMP-2, were evaluated by western blot. Data are reported as means±SD. **P<0.01 (ANOVA).

**Figure 4:**
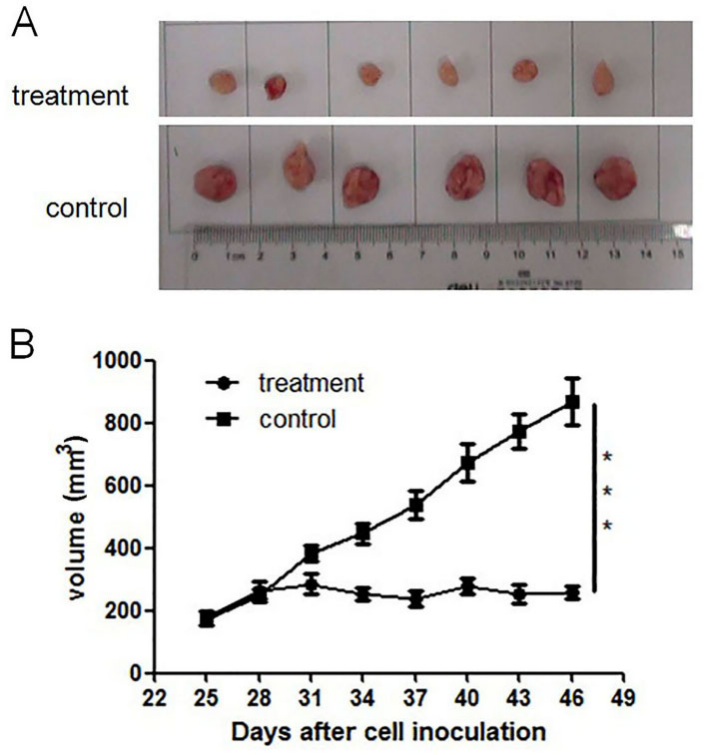
Inhibitive effects of ethanol extracts of *Antrodia cinnamomea* (EEA) on tumorigenesis *in vivo*. **A** and **B**, Nude BALB/c mice were inoculated with SAS cells and treated with EEA when tumors were palpable. Tumor growth was significantly inhibited by EEA administration. Data are reported as means±SD. ***P<0.001 (ANOVA).

**Table 1:** Antibodies used in the study.

Antibody	Cat No.	Dilution
GAPDH	8848	1:2000
Cleaved PARP	5625	1:1000
Cleaved caspase-9	9505	1:1000
MMP-2	40994	1:1000
MMP-9	13667	1:1000
TIMP-1	8946	1:1000
TIMP-2	5738	1:1000
Anti-rabbit IgG, HRP-linked antibody	7074	1:500
Anti-mouse IgG, HRP-linked antibody	7076	1:500

MMP: matrix metalloproteinase; TIMP: tissue inhibitors of metalloproteinase.

### Inhibition of invasion and migration by EEA

Results of wound healing assays indicated an average migration rate of 78.7% in the control group, while average migration rates were 65.3, 40.0, and 7.0% in groups treated with 0.5, 12.5, and 25 μg/mL EEA, respectively (Figure 2A and B). All these demonstrated that treatment of SAS with EEA for 36 h retarded cell migration ability in a dose-dependent manner. Data of transwell assay showed that, on average, 125 invasive cells per field were observed in control, and 72, 54, and 43 in the groups treated with 0.5, 12.5, and 25 μg/mL EEA, respectively (Figure 3A and B). Transwell assay illustrated that the number of invasive cells was significantly reduced by EEA treatment in a concentration-dependent manner. Matrix metalloproteinases (MMPs) and tissue inhibitors of metalloproteinase (TIMP) are the most studied factors that directly influence the invasion and migration capacities of tumor cells. As shown in Figure 3C, a dose-dependent decrease in expressions of MMP-2 and MMP-9 were observed in cells treated with EEA (0.5 to 25 μg/mL), while TIMP-1 and TIMP-2 expressions were not affected. Based on the above data, it can be proposed that EEA was an effective inhibitor of migration and invasion of SAS partially by decreasing expression of MMP-2 and MMP-9.

### Anti-tumorigenic ability of EEA

Based on the observed effects of EEA on SAS *in vitro*, we determined its impact on tumorigenesis *in vivo*. Tumor growth was significantly slowed down in mice treated by EEA compared to control (Figure 4A and 4B), indicating that EEA treatment significantly inhibited tumorigenesis *in vivo*.

## Discussion

Head and neck squamous cell carcinoma is globally recognized as one of the most common malignancies with an increasing incidence rate (3,18). Patients suffering from malignancies often experience recurrence or metastasis after treatments such as surgery, radiation therapy, chemotherapy, and targeted therapy (19,20), and clinical outcomes remain unsatisfactory (21). *Antrodia cinnamomea* has been commonly used as food for its diverse benefits for health, including anti-inflammatory (15), antioxidant, liver protection (22), and anticancer (7). Here we explored the potential application of *Antrodia cinnamomea* in treatment of head and neck squamous cell carcinoma.

Tumor cell proliferation and migration are mostly responsible for cancer progression. This study investigated the effects of EEA on proliferation and migration of HNSCC cells, and we found that EEA might inhibit proliferation and migration of SAS in a dose-dependent manner. Meanwhile, advanced experiments were conducted to briefly determine the mechanisms of the EEA-induced impact on SAS. We observed that cellular levels of cleaved caspase-9 and cleaved PARP were upregulated in cells treated with increasing doses of EEA, implying that provoking cell apoptosis was one of the mechanisms mediating the effect of EEA on proliferation. Recent study demonstrated that EEA could induce cell cycle arrest of lung cancer cells, thus leading to proliferation inhibition (23).

Tumor metastasis is a complicated process involving diverse types of proteins, of which the most well studied are MMPs and TIMP. In our study, we found that MMP-2 and MMP-9 expressions were decreased by administration of EEA while TIMP-1 and TIMP-2 were not affected. A study focusing on lung cancer also reported that extracts of *Antrodia cinnamomea* decreased expression of MMP-2 and MMP-9 as well as their activities, which was ascribed to dysregulation of ERK, JNK, p38, and PI3K/Akt signaling pathways (7). A constituent isolated from *Antrodia cinnamomea* was confirmed to be an anti-metastatic compound mostly via reducing expression of MMP-2 and MMP-9 and enhancing E-cadherin and TIMP-1 expression (24).

Natural products extracted from plants or microorganisms are important sources for drug screening (22,25). Several compounds extracted from fruit bodies of *Antrodia cinnamomea* have been found to efficiently influence tumor progression both *in vivo* and *in vitro*. For example, YMGKI-1, purified from natural products of *Antrodia cinnamomea*, was shown to effectively decrease cell viability of HNSCC cells and successfully inhibit tumorigenicity of SAS (14). Antcin-H, a type of triterpene isolated from *Antrodia cinnamomea*, was reported to inhibit proliferation and migration of human renal carcinoma 786-O cells by targeting MMPs and TIMPs (10). Furthermore, recent articles (12,13,16) revealed that EEA enhances chemo-sensitivity of several carcinomas, indicating that EEA can be used as adjuvant treatment combined with conventional chemotherapy. Several investigations have already shown the synergistic effects of EEA combined with cisplatin (16), sorafenib (12), and tamoxifen (13). The anti-tumor effect of EEA has been confirmed in a randomized controlled study, although the synergistic effects of EEA combined with chemotherapy were not observed (26). It has been proposed that cancer stem-like cells were involved in tumor initiation and occurrence of chemoradiotherapy resistance, responsible for compromised clinical outcomes of conventional therapies (2,27,28). Su et al. (17) reported that extracts of *Antrodia cinnamomea* sensitize cancer stem-like cells including HNSCC to chemoradiotherapy. Collectively, extracts of *Antrodia cinnamomea* are an important source for new drug discovery.

We conclude that this study comprehensively verified the inhibitive effects of EEA on cell proliferation, invasion, and migration of HNSCC *in vitro* and *in vivo*, and the potential molecular mechanism mediating the observed effects. These findings provide the basis for further study of the application of EEA as an effective candidate for cancer treatment.
